# Suppressed N_2_O formation during NH_3_ selective catalytic reduction using vanadium on zeolitic microporous TiO_2_

**DOI:** 10.1038/srep12702

**Published:** 2015-08-03

**Authors:** Seung Gwan Lee, Hyun Jeong Lee, Inhak Song, Seunghee Youn, Do Heui Kim, Sung June Cho

**Affiliations:** 1 Department of Applied Chemical Engineering, Chonnam National University, Yongbong 300, Buk-gu, Kwangju, 500-757, Korea; 2 School of Chemical and Biological Engineering, Institute of Chemical Processes, Seoul National University, 1 Gwanak-ro, Gwanak-gu, Seoul 151-744, Korea

## Abstract

Emission of N_2_O from mobile and off-road engine is now being currently regulated because of its high impact compared to that of CO_2_, thereby implying that N_2_O formation from the exhaust gas after-treatment system should be suppressed. Selective catalytic reduction using vanadium supported TiO_2_ catalyst in mobile and off-road engine has been considered to be major source for N_2_O emission in the system. Here we have demonstrated that vanadium catalyst supported on zeolitic microporous TiO_2_ obtained from the hydrothermal reaction of bulk TiO_2_ at 400 K in the presence of LiOH suppresses significantly the N_2_O emission compared to conventional VO_x_/TiO_2_ catalyst, while maintaining the excellent NO_x_ reduction, which was ascribed to the location of VO_x_ domain in the micropore of TiO_2_, resulting in the strong metal support interaction. The use of zeolitic microporous TiO_2_ provides a new way of preparing SCR catalyst with a high thermal stability and superior catalytic performance. It can be also extended further to the other catalytic system employing TiO_2_-based substrate.

Ever increasing demand for the reduction of greenhouse gas results in the more stringent regulation on its emission and also the corresponding research and development to capture or convert into inert molecule^1^. Compared to that of a major greenhouse, CO_2_, N_2_O has a high greenhouse gas effect up to 300 times^2^. Therefore, the impact of N_2_O emission can be comparable to that of CO_2_ though the emission concentration of N_2_O is relatively low. Most recent diesel engine emission regulation is now started to include N_2_O because of its high impact and stability in stratosphere^3^. For diesel engine emission control under lean condition, urea SCR (selective catalytic reduction) system is *the state of art technology* for the reduction of NO_x_ in most engine companies^4^. Under lean condition where the air to fuel ratio is far beyond the stoichiometric condition, the N_2_O formation can be suppressed readily while the system has been maintained under oxidizing condition. However, the emission of N_2_O from diesel engine can be increased when the reducing agent for NO_x_ is introduced in the SCR system following the reactions, such as 2NH_3_ + 2NO + O_2_ = N_2_O + N_2_ + 3H_2_O, 2NH_3_ + 2O_2_ = N_2_O + 3H_2_O and NH_4_NO_3_ = N_2_O + 2H_2_O^3^. The former two reactions were believed to be the major pathway for N_2_O formation in which bimolecular reaction can occur.

For NO_x_ abatement, VO_x_ catalyst supported on TiO_2_ has been used widely in most diesel engines^5^,^6^,^7^,^8^. There are numerous investigations on the improvement of the catalytic performance using additives such as Ce or W and also using peculiar TiO_2_ synthesized using sol-gel method or organic or inorganic templating method^9^,^10^,^11^,^12^,^13^,^14^,^15^. However, VO_x_ supported on TiO_2_ of relatively low surface area is *the state of art technology* catalyst. Indeed the current VO_x_/TiO_2_ catalyst emits N_2_O when the reducing agent is present in the stream. The current emission level of N_2_O is 50 mg per mile, which also depends on the catalyst composition and the system configuration such as diesel oxidation catalyst-selective catalytic reduction-diesel particulate filter^3^. The N_2_O emission characteristics of the VO_x_/TiO_2_ catalyst should be improved under reducing condition. For this purpose, the economically viable catalyst has to be developed in near future.

It has been demonstrated that the hydrothermal conversion of commercially available TiO_2_ in the presence of alkaline hydroxide produces unique TiO_2_ structures differently depending on the species of alkaline hydroxide^16^,^17^,^18^,^19^. Recently, the addition of LiOH, NaOH and KOH to the hydrothermal medium was reported to be resulted in the formation of zeolitic microporous TiO_2_, nanotube and nanorod, respectively^19^,^20^,^21^, which seems to be a cost effective process. The obtained microporous nanocrystalline TiO_2_ showed large surface area of 250 m^2^g^−1^ with the pore volume of 0.15–0.20 ccg^−1^, which was similar to those of zeolites and also suitable for catalyst preparation.

The formation mechanism of TiO_2_ nanotube by hydrothermal synthesis in the presence of NaOH has been studied extensively but it is not clarified yet^16^,^22^. Initial work on the formation mechanism of TiO_2_ nanotube suggested that the nanotube is obtained with an acid washing and subsequent Na^+^ ion exchange after the formation of amorphous TiO_2_ during hydrothermal reaction between NaOH and bulk TiO_2_^16^,^20^,^22^,^23^,^24^. The other mechanism proposed that the bond breaking of 3-dimensional TiO_2_ structure formed layered 2-dimensional structure and finally 1-dimensional nanotubes through sheet folding mechanism^22^. The removal of Na^+^ cation from the nanotube also deteriorates readily the thermal stability when it is heated at high temperature.

However, the use of zeolitic microporous TiO_2_ prepared from alkaline condition has not explored yet. Thus, it is interesting to know whether the zeolitic microporous TiO_2_ has a high thermal stability or not and also is suitable for catalytic application as a substrate. For the first time in the present work, the zeolitic microporous nanocrystalline TiO_2_ has been demonstrated as a catalyst support for VO_x_ over selective catalytic reduction of NO_x_ using ammonia in order to decrease the N_2_O formation.

## Results

**Figure 1:**
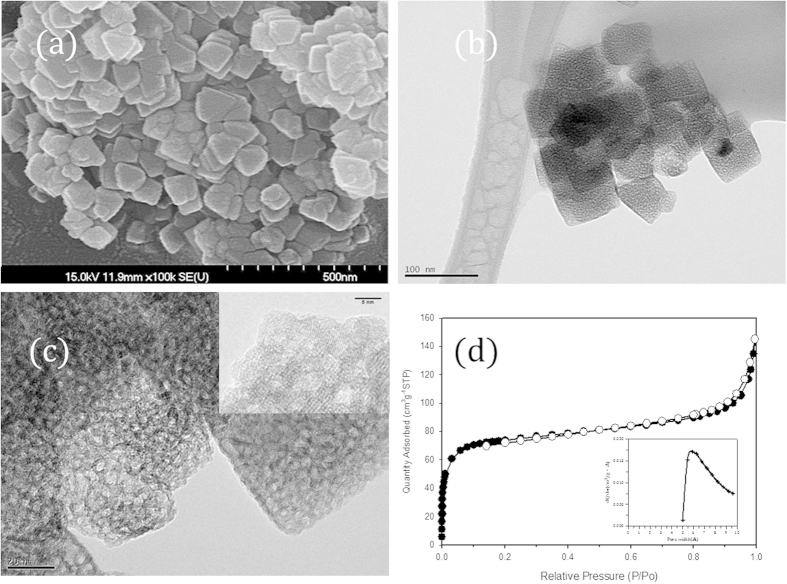
Morphology of the zeolitic microporous TiO_2_. (**a**) Scanning electron micrographs, **(b,c)** transmission electron micrographs and (**d**) the argon adsorption-desorption isotherm of the zeolitic microporous TiO_2_ with the pore size distribution (inset) obtained from Horvath-Kawazoe method. The solid and open symbols indicate the adsorption and desorption branch, respectively.

Figure 1 shows the scanning electron micrographs and transmission electron micrographs of the zeolitic microporous TiO_2_ after the hydrothermal treatment. The morphology of the particle after the hydrothermal conversion contained sharp edges, suggesting the formation of the well-crystallized TiO_2_, which was consistent with the literature^19^,^20^,^21^. The obtained TiO_2_ had the typical argon adsorption-desorption isotherm consistent with the Langmuir isotherm type containing micropore mostly where the micropore area estimated from *t*-plot was ~200 m^2^g^−1^, corresponding to 80% of the total surface area of which the pore size was estimated to be ~7 Å. The corresponding surface area and pore volume were controlled to be 250 ± 20 m^2^g^−1^ and 0.20 ± 0.05 ccg^−1^, respectively, depending on the hydrothermal reaction condition. Also, the presence of the mesopore was observed above *P*/*P*_0_ > 0.9 but its portion can be decreased with the increase of hydrothermal reaction time. Such mesopore formation was also shown clearly in Fig. 1(c) where the mesopore was formed with several interconnecting crystalline TiO_2_ frameworks of which the thickness was 3–4 nm.

Therefore, the results of the transmission electron micrograph observation supported the corresponding unique TiO_2_ structure containing the micropore and also the mesopore of which the size was 5–7 nm, which was consistent with the result of argon and nitrogen adsorption-desorption measurement.

The hydrothermal conversion of the bulk TiO_2_ into zeolitic microporous TiO_2_ in the presence of LiOH seems not to follow the sheet folding mechanism like TiO_2_ nanotube. The intercalation of Li^+^ ion into the TiO_2_ structure leads to the formation of the Li^+^-O-Ti bond similar to that of Na^+^ case, resulting in the partial delamination of the TiO_2_ layer where the interaction between the layers is high enough to induce the combination of the corresponding layers^25^,^26^.

**Figure 2:**
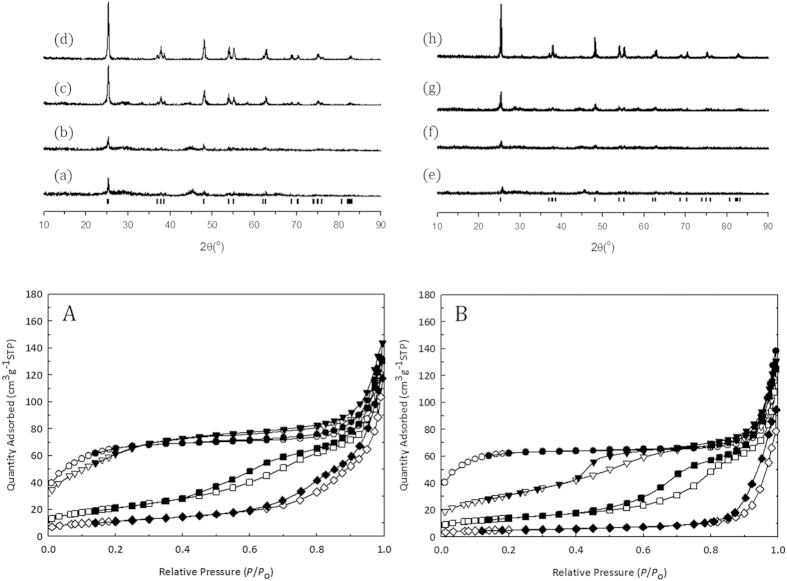
Phase and surface properties of the zeolitic microporous TiO_2_. X-ray powder diffraction pattern of the zeolitic microporous TiO_2_ heated at (**a,e**) 673 K, (**b,f** ) 773 K, (**c,g**) 873 K and (**d,h**) 973 K for 4 h, respectively, in ambient air with (upper right panel) and without saturated water (upper left panel). The tick mark corresponded to anatase phase. Nitrogen adsorption-desorption isotherm for the zeolitic microporous TiO_2_ heated at (○,•) 673 K, (▽,▾) 773 K, (□,■) 873 K, and (◇, ◆) 973 K, respectively, in ambient air with (lower left panel, A) and without saturated water (lower right panel, B). The open and solid symbols indicate the adsorption and desorption branch, respectively.

The thermal stability of the zeolitic microporous TiO_2_ under ambient condition either with the presence or absence of the saturated water was also investigated using the X-ray diffraction pattern (XRD) and N_2_ adsorption and desorption isotherms as a function of heating temperature. Nearly up to 773 K, the microporous structure was retained as evidenced from the results of the nitrogen adsorption-desorption isotherm for the sample shown in Fig. 2 even though the microporous structure was collapsed significantly with the increase of temperature in the presence of water. The XRD of the sample also showed that the crystalline anatase structure appeared to be the major phase when the samples were heated above 773 K. The combined results from XRD and nitrogen adsorption-desorption isotherm confirmed the transformation of microporous TiO_2_ to macroporous TiO_2_. The moderate thermal stability of the zeolitic microporous TiO_2_ due to the crystal growth into anatase in the presence of water may limit the catalytic application at higher temperature. However, many catalytic applications including photocatalysis or solar energy harvesting can adopt the present zeolitic microporous TiO_2_^21^.

**Figure 3:**
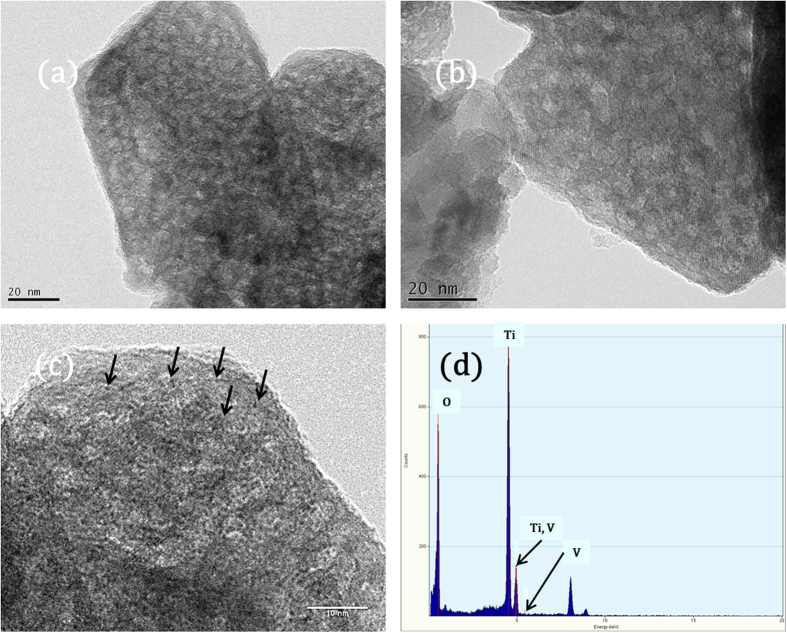
VO_x_ encapsulated in the zeolitic microporous TiO_2_. Transmission electron micrographs of the microporous TiO_2_ containing 5 wt% heated at (**a**,**c**) 673 K and (**b**) 773 K, respectively. The elemental analysis in (**d**) showed the presence of V.

VO_x_ was supported onto the corresponding unique TiO_2_ structure following the procedure reported in the literature^27^,^28^,^29^,^30^,^31^,^32^. However, the supporting VO_x_ catalyst up to 5 wt% resulted in the lower surface area of 105 m^2^g^−1^ with 0.19 ccg^−1^ because of the high calcination temperature at 773 K though the sample still contained large surface area. These textural properties were maintained before and after the catalytic reaction measurement. The TEM observation of VO_x_ incorporated TiO_2_ as shown in Fig. 3 suggested that most VO_x_ particle are located inside the pores of the zeolitic microporous TiO_2_, ~7 Å without changing the corresponding morphologies. Thereby, the VO_x_ particle was observed clearly as a spot in Fig. 3(c) while there was no particle on the external surface of the TiO_2_ though the elemental analysis showed the presence of V in the same region as shown in Fig. 3(d), which can be beneficial for the catalytic reaction. Further, increasing V content in the present also did not alter the location of V.

**Figure 4:**
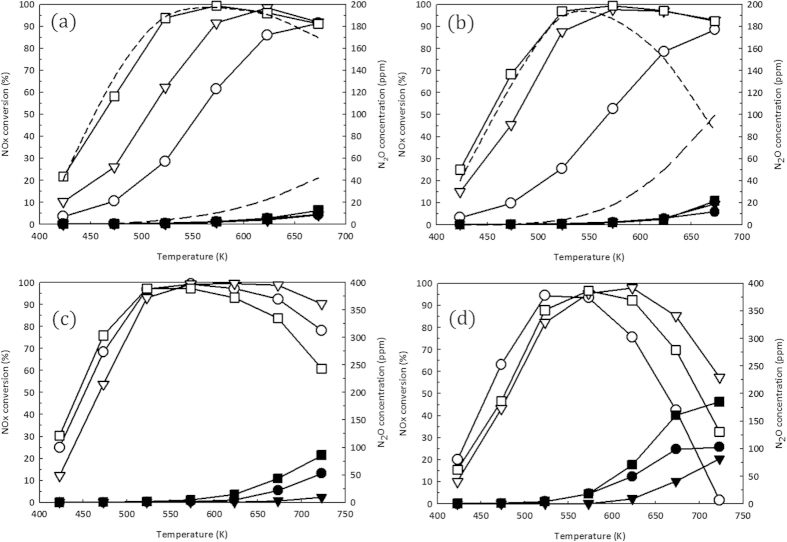
SCR activity of VO_x_ encapsulated in the zeolitic microporous TiO_2_. Catalytic activity of V/TiO_2_ catalyst calcined at (**a**) 673 K and (**b**) 773 K for over NO_x_ reduction using ammonia: (○,•) 1wt%, (▽,▾) 3 wt% and (□,■) 5 wt%. The long and short dashed lines also represent the N_2_O concentration and NO_x_ conversion from conventional V/TiO_2_, respectively. The open and solid symbols were corresponded to NOx conversion and N_2_O formation, respectively. The dashed line was the catalytic performance of commercial V/TiO_2_. The reactant consisting of 500 ppm NO, 500 ppm NH_3_, 2% O_2_ balanced with N_2_ was flowed through the V/TiO_2_ catalyst bed containing 0.15 g at *GHSV* = 40,000 h^−1^. The catalytic activity of (**c**) 5 wt% V/TiO_2_ catalyst and (d) conventional V/TiO_2_ catalyst calcined at 773 K were measured in the different reaction conditions : (○,•) dry reaction condition, (▽,▾) wet reaction condition and (□,■) dry reaction condition with hydrothermal aging at 773 K for 12 h in the presence of 5% water. In order to achieve the wet condition, the reactant consisting of 500 ppm NO, 500 ppm NH_3_, 2% O_2_ and 3% H_2_O balanced with N_2_ was flowed through the V/TiO_2_ catalyst bed at *GHSV* = 40,000 h^−1^.

Figure 4 shows the catalytic performance of the V/TiO_2_ catalyst after the calcination at different temperatures also under various reaction conditions. Increasing V content in the catalyst improved the catalytic performance of the NO_x_ reduction comparable to that of conventional V/TiO_2_ catalyst containing 5 wt% V over the whole reaction temperature range while the N_2_O formation was much lower than that of conventional catalyst. The N_2_O formation from the present V/TiO_2_ was increased with the increase in the temperature where NH_3_ was combined with NO_x_ to produce N_2_O. The effect of the calcination temperature was also pronounced to increase the N_2_O formation at high temperature but the N_2_O formation was still lowered than that of the conventional catalyst by more than 80%. Also, the V/TiO_2_ catalyst prepared from the microporous TiO_2_ resulted in the superior catalytic performance over the SCR reaction both in the presence of water in the reactant stream and after the aging in the presence of water at 773 K for 12 h, as shown in Fig. 4(c,d). In the presence of water in the stream the low temperature catalytic activity was decreased while the high temperature catalytic activity was increased slightly because of competitive adsorption of NH_3_ and H_2_O suppressing the NH_3_ oxidation.

Under the present condition, the main reaction for N_2_O formation is believed to be 2NH_3_ + 2NO + O_2_ = N_2_O + N_2_ + 3H_2_O following the literature^3^. The catalytically active VO_x_ inside the pore, ~7 Å was believed to have a strong metal support interaction with TiO_2_, resulting the smaller VOx particle size as referred from Fig. 3. Thus, the strong metal-support interaction between TiO_2_ and VOx led to the formation of Bronsted acid site with high strength, which is beneficial for selective catalytic reduction of NO by NH_3_. The N_2_O formation can be suppressed up to ~80% because of the increased Bronsted acidity of the VOx small particle in the microporous zeolitic TiO_2_ where the superior SCR activity can be maintained as illustrated in Fig. 4(c). This result was partly consistent with the increased N_2_O formation on the V/TiO_2_ catalyst when the catalyst deteriorates because of the sintering^3^. Also, it was possible to include NH_3_ oxidation by O_2_ as potential pathway for the following N_2_O formation reaction: 2NH_3_ + 2O_2_ = N_2_O + 3H_2_O where the catalyst deactivation was severe like the commercial V/TiO_2_ catalyst in the presence of water or after hydrothermal aging. One possibility to explain the superior catalytic performance V supported on zeolitic microporous TiO_2_ over the SCR reaction was that the growth of the vanadium oxide particle size can be limited due to the pore size, implying the encapsulation of vanadium oxide particle surrounded by TiO_2_ matrix.

We have demonstrated that the zeolitic microporous TiO_2_ with moderate thermal stability can be prepared from the simple hydrothermal conversion from commercially available bulk TiO_2_ of low grade, 98% or lower in the presence of LiOH at 400–440 K, which can be scaled up easily for industrial process. The obtained zeolitic microporous nanocrystalline TiO_2_ contains the micropore up to 80% referred from the *t*-plot method. For the first time, it was proved that that the supporting VO_x_ into such zeolitic microporous TiO_2_ resulted in the high NO_x_ reduction activity with lower N_2_O formation, which was ascribed to the location of catalytically active VO_x_ particles in the microporous TiO_2_, resulting the strong metal-support interaction and consequently the increased Bronsted acidity. Therefore, the zeolitic microporous TiO_2_ has potential as a substrate for the SCR reaction below 773 K while the thermal stability of the microporous TiO_2_ was retained.

## Methods

### Synthesis of zeolitic microporous TiO_2_

TiO_2_ anatase (Aldrich, 98%) of 2–8 g was added to the solution containing 10 M or more LiOH in the Teflon lined autoclave for hydrothermal heating at 400–440 K for 72 hr under rotating condition at 40 rpm. After cool down to room temperature, the slurry was neutralized with 0.1 N HCl under stirring for 6 hr. The solution was filtered and washed with deionized water thoroughly. The acidification and filtration was repeated three times to remove the residual trace metal hydroxides. The obtained product was dried at 330 K in an oven and calcined under flowing oxygen at 673 K for 4 h. The inductively coupled plasma analysis of the obtained sample showed that the residual Li was ~6 ppm level, indicating the complete removal of Li^+^ by the neutralization and subsequent thorough washing. The scale up to ~100 g per batch was also demonstrated to give the same textural properties.

### Preparation of VO_x_ in zeolitic microporous TiO_2_

All catalysts were prepared by applying wet impregnation of vanadium precursor solution on titania. Ammonium metavanadate (99%, Sigma Aldrich) was dissolved in diluted oxalic acid solution (0.5 M) to produce the solution of vanadium precursor. Anatase TiO_2_ powder (DT-51 Millennium Chemicals) was used as support to prepare the conventional catalyst containing 5 wt% V. The samples with 1 wt%, 3 wt% and 5 wt% V_2_O_5_ loading on TiO_2_ were prepared. After impregnation process in a rotary evaporator, catalysts were dried and then calcined at 673 K or 773 K for 4 h in air.

### SCR activity measurement of VO_x_ in zeolitic microporous TiO_2_

SCR activity was measured in a fixed-bed quartz tubular reactor. Catalysts were sieved to 300–500 μm in diameter then loaded in the reactor. 500 ppm NO, 500 ppm NH_3_, 2% O_2_ and balanced with N_2_ were introduced as reactants. In order to examine the catalytic activity in the presence of water, the reactant containing 500 ppm NO, 500 ppm NH_3_, 2% O_2_, 3% H_2_O balanced with N_2_ was used. The catalyst was further aged in the presence of 10% O_2_, 5% H_2_O balanced with N_2_ at 500 ^o^C for 12 h before catalytic reaction.

Space velocity of inlet gas was maintained to be 40,000 h^−1^. We raised reaction temperature from 423 K to 673 K by 50 K. NOx concentration of outlet gas by using NOx chemiluminescence analyzer (Model 42i High level, Thermo Scientific). Also, Fourier Transform Infrared (FT-IR) spectroscopy was applied to observe the N_2_O concentration in the gas. We used the average data of 16 scans at a resolution of 1.0 cm^−1^. A Nicolet 6700 (Thermo Scientific) with 2 m gas analysis cell heated to 120 °C to exclude the effect of H_2_O, was used for gas phase analysis.

## Additional Information

**How to cite this article**: Lee, S. G. *et al.* Suppressed N_2_O formation during NH_3_ selective catalytic reduction using vanadium on zeolitic microporous TiO_2_. *Sci. Rep.* **5**, 12702; doi: 10.1038/srep12702 (2015).
